# Deep learning of mutation-gene-drug relations from the literature

**DOI:** 10.1186/s12859-018-2029-1

**Published:** 2018-01-25

**Authors:** Kyubum Lee, Byounggun Kim, Yonghwa Choi, Sunkyu Kim, Wonho Shin, Sunwon Lee, Sungjoon Park, Seongsoon Kim, Aik Choon Tan, Jaewoo Kang

**Affiliations:** 1 Department of Computer Science and Engineering, Korea University, Seoul, South Korea; 2 Interdisciplinary Graduate Program in Bioinformatics, Korea University, Seoul, South Korea; 3 Translational Bioinformatics and Cancer Systems Biology Laboratory, Division of Medical Oncology, Department of Medicine, University of Colorado Anschutz Medical Campus, Aurora, CO 80045 USA

**Keywords:** Deep learning, Convolutional neural networks, Information extraction, Text mining, NLP, BioNLP, Mutation, Precision medicine

## Abstract

**Background:**

Molecular biomarkers that can predict drug efficacy in cancer patients are crucial components for the advancement of precision medicine. However, identifying these molecular biomarkers remains a laborious and challenging task. Next-generation sequencing of patients and preclinical models have increasingly led to the identification of novel gene-mutation-drug relations, and these results have been reported and published in the scientific literature.

**Results:**

Here, we present two new computational methods that utilize all the PubMed articles as domain specific background knowledge to assist in the extraction and curation of gene-mutation-drug relations from the literature. The first method uses the Biomedical Entity Search Tool (BEST) scoring results as some of the features to train the machine learning classifiers. The second method uses not only the BEST scoring results, but also word vectors in a deep convolutional neural network model that are constructed from and trained on numerous documents such as PubMed abstracts and Google News articles. Using the features obtained from both the BEST search engine scores and word vectors, we extract mutation-gene and mutation-drug relations from the literature using machine learning classifiers such as random forest and deep convolutional neural networks.

Our methods achieved better results compared with the state-of-the-art methods. We used our proposed features in a simple machine learning model, and obtained F1-scores of 0.96 and 0.82 for mutation-gene and mutation-drug relation classification, respectively. We also developed a deep learning classification model using convolutional neural networks, BEST scores, and the word embeddings that are pre-trained on PubMed or Google News data. Using deep learning, the classification accuracy improved, and F1-scores of 0.96 and 0.86 were obtained for the mutation-gene and mutation-drug relations, respectively.

**Conclusion:**

We believe that our computational methods described in this research could be used as an important tool in identifying molecular biomarkers that predict drug responses in cancer patients. We also built a database of these mutation-gene-drug relations that were extracted from all the PubMed abstracts. We believe that our database can prove to be a valuable resource for precision medicine researchers.

**Electronic supplementary material:**

The online version of this article (10.1186/s12859-018-2029-1) contains supplementary material, which is available to authorized users.

## Background

Precision medicine aims to deliver personalized treatment to individual patients based on their genomic profiles. Identifying molecular biomarkers such as genes with specific mutations to predict the efficacy of a drug in cancer patients is important for the advancement of precision medicine. For example, the BRAF V600E mutation in melanoma patients can be used to predict response to BRAF inhibitors such as vemurafenib [1]. However, BRAF V600E has no predictive value for BRAF inhibitors in colorectal cancer patients [2]. Thus, understanding the relations between genes, mutations and drugs in a specific context (e.g. disease) is crucial for the development of molecular biomarkers.

The systematic characterization of cancer cell lines using next-generation sequencing coupled with high-throughput drug screening has generated rich experimental data for pharmacogenomics. Large-scale research projects such as Genomics of Drug Sensitivity in Cancer (GDSC) [3], Cancer Cell Line Encyclopedia (CCLE) [4] and Cancer Therapeutics Response Portal (CTRP) [5] provide gene-mutation-drug relations for the advancement of personalized medicine. Also, databases such as ClinVar [6], My Cancer Genome [7], MD Anderson Personalized Cancer Therapy Knowledgebase [8] contain gene-mutation-drug relations extracted from manually curated literature on clinical studies. Unfortunately, manually curating all gene-mutation-drug relations is infeasible due to the large number of on-going sequencing projects and the fast-growing volume of research articles reporting new relations. Computational methods that automatically extract gene-mutation-drug relations from the literature are urgently needed to assist in the curation process.

The named entity recognition (NER) process, which is a necessary process of automated information extraction methods, involves finding biomedical entities in text. NER identifies mutations, genes, diseases, and drug names in text. Many NER tools have been developed to identify different entities in text; for example, tmVar [9], EMU [10], and MutationFinder [11] identify mutations; BANNER [12] and GNormPlus [13] identify genes; and ChemSpot [14] and tmChem [15] identify drugs. BEST Biomedical Entity Extractor [16, 17] is a dictionary-based NER tool that identifies gene, disease, drug and cell line names. However, identifying the relations between entities (e.g., gene-mutation, gene-drug, mutation-drug, or gene-mutation-drug) remains a difficult task in NER.

Efforts have been made to develop methods that can capture relations between entities based on co-occurrence information in text [10, 18, 19]. Finding relations using co-occurrence information usually obtains high recall but low precision. To fix the low precision problem, some researchers added additional methods to their co-occurrence based models. For example, HiPub [19] shows the relations between entities using not only sentence-level co-occurrence but also information from external databases such as PharmGKB [20], DrugBank [21], and so on. Doughty et al. [10] extracted gene/protein and mutation names from texts and mapped them using a protein sequence filter in addition to co-occurrence information. Their gene-filtering tool checks amino acid sequences from NCBI RefSeq and compares them with wild type amino-acid information containing mutation names. However, this gene-filtering tool can find associated gene names only for amino-acid level mutations (e.g., p.V600E), and not DNA-level mutations (e.g., c.1799 T > A). Burger et al. expanded the former result of Doughty et al. by combining the automated relation extraction method with crowdsourcing [22]; however, crowdsourcing is still expensive and time consuming compared with fully automated methods.

The other group of methods used pre-defined rules with trigger words to find relations between entities. SNPshot [23] used sentence-level co-occurrence and pre-defined keywords to identify relations between entities. Mahmood et al. used a series of natural language processing (NLP) modules with part-of-speech tagging to find syntactic structures and specific pre-defined keywords in sentences containing mutations [24]. Using these features, they made several rules for finding relations between mutations, genes and diseases at the sentence level. However, these methods using pre-defined rules and keywords require the expensive labor of domain experts to generate rules and to find keywords that signify relations between entities. Also, the pre-defined rules have the risk of overfitting and they may be unsuitable for newly published articles containing new terms.

To overcome these limitations, some groups used machine learning to find relations between entities. Mallory et al. [25] employed DeepDive to extract gene-gene interactions from sentences and achieved reasonable precision on a large-scale literature test set. Singhal et al. [26] used a machine learning approach to identify mutation-gene-disease relations in the literature. They extracted simple general features such as the distance between a mutation and a disease, frequency of disease occurrence, and frequency of co-occurrence of mutation-disease pairs. They also used the sentiment scores between a mutation and a disease when they appeared in the same sentence. Using these features, they trained a decision tree classifier, and achieved better performance than state-of-the-art approaches used for finding gene-disease associations. Moreover, since this approach is independent of specific sentence structures, it can be used to identify other associations such as mutation-drug associations. We used the approach proposed by Singhal et al. as the baseline in this research because it not only outperforms all the other relation extraction methods but also is the only method that can be applied to the mutation-drug relation extraction task.

For methods that automatically extract mutation-gene and mutation-drug relation information, we have recently developed BRONCO which is a manually curated mutation-gene-disease-drug relation corpus [27]. In the process of constructing BRONCO, we observed that the curation accuracy of the domain experts was higher than that of the non-domain experts. As also shown in the study by Poux et al., domain experts use their background knowledge for curation, which helps improve the accuracy of the curation results [28]. For example, when domain experts who have extensive knowledge on melanoma annotate a text and see V600E, melanoma (disease), and BRAF (gene) in the text of an article, they can easily map V600E to the disease name and gene name. Domain experts are also very familiar with the descriptive terms that imply the associations between entities and that help them understand sentences faster and more accurately. However, if curators have little or no background knowledge or are unfamiliar with the terms in a text, it is more difficult for them to identify the relations in the text and thus have a higher chance of missing important information. Based on this observation, we believe automated methods can also perform better with background domain knowledge.

In this research, we built a machine learning classification models combined with two additional novel methods for using all the PubMed articles as our background domain knowledge, as domain experts have similarly done.

We used a deep learning classifier as one of the machine learning models. Text mining using deep learning has advantages especially in feature generation [29]. To extract specific information from documents using traditional text mining methods, an extremely time-consuming feature engineering process by domain experts is required in most cases. Furthermore, when the target information to extract is described in many ways in documents, it is difficult to select or generate specific features to extract that information. However, deep learning based text mining methods do not require any process or require a simpler feature generation process; instead, they can automatically extract features. In our variant-entity relation extraction task, many of the relations have different forms and some of them are described in a complicated way in documents. We thought a deep learning method would be effective for this task. We used deep convolutional neural network (CNN) which is a deep learning technique that uses multiple layers of neurons and convolutional layers for classification. We chose to use CNN for the following two reasons: 1) recently, good results were obtained in relation extraction tasks using CNN [30], 2) and CNN could be more practical than Recurrent neural network (RNN) from a computational perspective because RNN has connections that form a cycle which makes it parallel-processing unfriendly [31].

We used the query result from an entity search engine built for PubMed abstracts, as features for machine learning classification. We also used pre-trained word2vec [32] word vectors that are constructed using all the PubMed abstracts for a deep convolutional neural network model. Using the entity search engine, the system can instantly find existing knowledge in all the articles in PubMed and utilize the information for curation. Word vectors are used to obtain information about terms used in PubMed articles. We demonstrate that our newly developed deep learning classifier achieves comparable results in identifying gene-mutation relations and achieves better results in identifying mutation-drug relations, compared with the method (baseline) by Singhal et al.

## Methods

### Overview

**Fig. 1:**
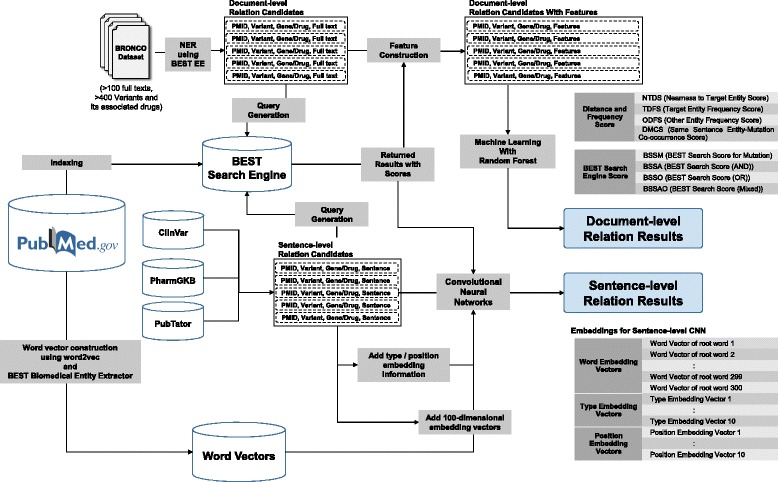
Overall workflow of the proposed methods

Figure 1 illustrates the overall workflow of the proposed mutation-entity extraction models using deep learning. Since the baseline model is based on finding mutation related entities in a document-level dataset, we designed two different models: a machine learning model using features constructed at the document-level, and a deep convolutional neural network model using features constructed at the sentence-level.

### Document/sentence level extraction – Problem definitions

We define the problems as document-level and sentence-level extraction. In document-level extraction, we generate all the possible combinations of relations between entities and classify them. For example, in a document, when the total number of unique mutation is m, and the total number of drugs (or genes) is n, all the possible m X n relations are the candidate relations. Our goal is building a machine learning model that classifies these relations into true and false groups. If a mutation-entity relation is true in any part of the document, the relations are considered as true. In this document-level extraction, even though the two entities are not in the same sentence, the relations are still in the candidate set. However, sentence-level extraction focuses on only the relations between entities at the single sentence level. In sentence-level extraction, we do not consider the frequency of the entities or the context of the whole text. Since document-level extraction uses more information, it can more easily classify relations than sentence-level extraction. However, sentence-level extraction can be more practical for real world use because it directly suggests the sentences that contain relations. At the sentence-level, when a mutation-entity relation is mentioned in the sentence, the relation is considered as true. For mutation-drug relations, both the drug-sensitive mutation or drug-resistant mutation relations are considered as true.

### Feature construction using BEST

#### Biomedical entity search tool (BEST)

BEST [16] is a biomedical entity search engine that works on all PubMed articles. For a user query, BEST returns a list of biomedical entities that are most related to the query. When a user inputs a query, BEST searches its index of all PubMed articles, and retrieves all the documents that contain the query. BEST also finds biomedical entities in the retrieved documents and ranks them using its scoring method. This returned list of entities with scores reflects how many times the input query and the entities co-occurred in PubMed articles, which is a very important clue that can be used to predict the associations between the query and the returned entities. For example, when a user inputs mutation “V600E” as the query, BEST returns “BRAF” and “melanoma” as its top gene and disease category results, respectively (see Table 1).

**Table 1:** BEST search result examples

	Drug category results	
Query	Top Result Entity	Score
T790 M	gefitinib	138.840
T790 M lung carcinoma	erlotinib	8.315
T790 M breast carcinoma	lapatinib	0.456
	Gene category results	
Query	Top Result Entity	Score
T790 M	EGFR	530.279
T790 M lung carcinoma	EGFR	21.874
V600E	BRAF	1589.055
G12D	KRAS	190.755

Although searching the entire PubMed corpus is challenging, BEST can instantly return a query result due to its efficient index structure. BEST uses an automatic update module to update itself daily with newly published articles in PubMed, which allows it to return the most up-to-date results. BEST can also process multiple-term queries to find the relations between the query entities. For example, as shown in Table 1, when the query is “T790 M lung carcinoma,” the top drug result returned is “erlotinib.” However, if the query is “T790 M breast carcinoma,” the top drug result is “lapatinib.” This multiple-query input enables us to find entities that are most closely related in a different context. Erlotinib is a well-known non-small cell lung cancer drug. It is widely known that patients who have the EGFR T790 M mutation are resistant to erlotinib.

As shown in Table 1, for the same query “T790 M lung carcinoma,” the score of the top result “erlotinib” is 8.315. However, “lapatinib” which is the top result of the second query “T790 M breast carcinoma” has a score of 0.456 only. On the other hand, the score of “gefitinib,” which is the top result of the query “T790 M,” is 138.84. Based on these results, we can assume that the T790 M mutation is closely related to gefitinib, and lung carcinoma with the T790 M mutation is slightly related to erlotinib. However, even though lapatinib is returned from the query “T790 M breast carcinoma,” the score is very low, which implies that lapatinib may not be closely related to T790 M. The details of the BEST scoring method are available in its online user guide [33].

#### BEST search engine scores as features

As explained in the previous section, BEST returns a list of entities with each entities’ search scores as the query result. We used these scores as features to find mutation-gene and mutation-drug relations. We used four different ways of querying BEST to obtain the result scores. First, we queried using only the normalized mutation name. For example, if BRONCO contains the mutation “Val600Glu,” we change it to “V600E” which is the most common form used to describe the mutation in the literature and is also the standard nomenclature suggested by HGVS [27, 34]. After entering this query, we obtained the result list of entities with their scores. This score is called BSSM. The second method uses not only the mutation itself but also the other biomedical entities that appear near the mutation to generate the query. For example, when we enter a query to find the relation between a mutation and a drug, we check all the biomedical entities such as gene names, disease names, and cell line names that appear in the same sentence. It is important to note that we do not use the entities of the same kind as the target entity. For example, if we are querying to find mutation-drug relations, we do not use any drug names for the query even though they appear in the same sentence.

**Fig. 2:**
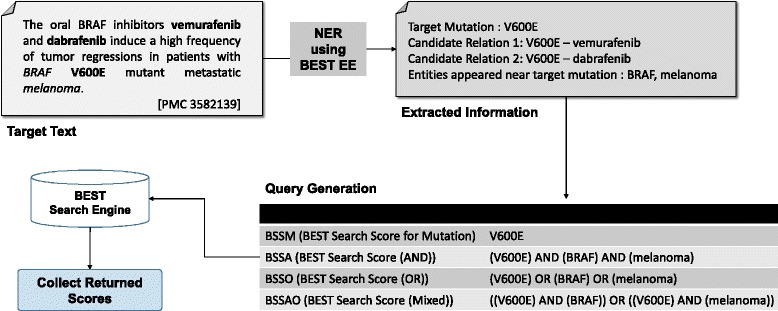
Query generation example of finding mutation-drug relations

We exclude the entities of the same kind as the target entity in the query because the same kind of entities adds noise rather than providing context information. From the sentence “In a randomized phase III study, dabrafenib showed prolonged progression-free survival compared with dacarbazine in patients with BRAF V600E metastatic melanoma [PMID 24769640],” we generate a query with V600E, BRAF and melanoma to obtain the score of dabrafenib from BEST’s search engine (score 78.427) and evaluate the dabrafenib-V600E relation. In this sentence, dacarbazine which is a drug, does not provide context information on the relation between V600E and dabrafenib. If we include dacarbazine in the query, we obtain a much lower score for dabrafenib (score 11.052) but a higher score for dacarbazine (21.550). If we include drugs in queries, it can distort the strength of target mutation-drug relations. We used three different methods to generate multiple entity queries containing “AND” or “OR,” and combined the results obtained from these multiple entity queries. Figure 2 illustrates an example of the BEST query process using these methods.

### Word vectors constructed from PubMed

Using word2vec [32], we constructed 300-dimensional word vectors trained on the PubMed dataset. Pyysalo et al. [35] made word vectors trained on PubMed data; however, multi-token words were not considered in their work. We believe that “non-small cell lung cancer” needs to be recognized as an entity rather than a simple list of four different words. For this reason, we first performed named-entity recognition on the multiple words and changed the multiple token biomedical terms to a single token term. For example, we converted “non-small cell lung cancer” to “non-small_cell_lung_cancer.” We trained our word vectors on all the 27 million PubMed abstracts. We obtained word vectors for more than 5 million words except stop words. We removed words with a frequency less than five from the word vectors before training the word vectors. Typically these low frequency words are removed when training word vectors [32] because they act as noise and require a considerable amount of time and computational resources. We used the Python implementation of the word2vec training method obtained from the Gensim word2vec tutorial [36]. We also used 300-dimensional word vectors that were trained on the Google News datasets [37].

### Distance and frequency scores as features

Singhal et al. [26] defined six features for determining the relations between mutations and diseases. Out of the six features, four of them are based on the distance between entities and the frequency of the entities. The Nearness to Target Disease Score (NTDS) represents the number of co-occurrences of a target disease and a mutation. The Target Disease Frequency Score (TDFS) denotes the frequency of the target disease. The Other Disease Frequency Score (ODFS) represents the frequency of the most frequent disease, except the target disease, in the document. The same sentence Disease-mutation Co-occurrence Score (DMCS) is a binary score that denotes whether a mutation and the disease nearest to the mutation are mentioned in the same sentence. We used these features as distance and frequency based features for our classification models.

### Dataset

#### BRONCO as a document-level evaluation dataset

BRONCO [27] is a biomedical entity relation oncology corpus that contains 108 full-text articles related to cancer and anti-tumor drug screening research. It contains information on more than 400 mutations and their associations with genes, diseases, drugs and cell lines. BRONCO is available at http://infos.korea.ac.kr/bronco/.

We generated all the possible mapping pairs using the BRONCO dataset. Given all the mutations in BRONCO, we found all the genes and drugs that appear in the same text, and generated all the candidate mutation-gene and mutation-drug relation pairs. All the gene and drug names in the text are identified using BEST entity extractor. Among these candidate relations, pairs in BRONCO are tagged as true, and others are tagged as false. By this process, we generated 9615 candidates with 277 positive mutation-gene relations, and 7658 candidates with 297 positive mutation-drug relations. Due to the imbalance in the positive-negative ratio of the dataset, we sampled the same number of positive-negative cases, and used these for our document-level evaluation dataset.

#### Mutation-gene relation sentence dataset using ClinVar and COSMIC

Deep learning requires a large dataset for training a model. For training, we generated a mutation-gene relation dataset. We first used PubTator [38] to compile a list of the PMIDs that contain at least one mutation and one gene name. PubTator provides the named-entity recognition results of biomedical entities such as genes, diseases, drugs and mutations in PubMed abstracts. Using PubTator data, we can find all the PubMed abstracts containing genes, drugs and mutations. We downloaded the bulk data from its FTP site and found the list of PMIDs that contain at least one mutation and one drug name. This process made it possible to look at only the abstracts that mutation exists rather than looking at all the 27 million PubMed abstracts. ClinVar [6] and COSMIC [39] provide files of mutation-gene-PMID mapping data. We used the abstracts obtained from PubTator to find sentences containing mutation-gene relations in specific PMIDs. We also used amino-acid sequences of genes from UniProt to filter erroneous gene-mutation relations, which is shown in EMU’s SEQ_Filter method [10]. All the sentences that passed these three steps of filtering are included for the positive training dataset. For the negative training dataset, we found sentences containing mutation-gene pairs that are not contained in the ClinVar or COSMIC databases; the SEQ_Filter method defines mutation-gene pairs as erroneous. Using this method, we obtained 4440 and 165,317 sentences for the positive and negative training datasets for mutation-gene relation sentence dataset, respectively.

#### Mutation-drug relation sentence dataset using PharmGKB

As deep learning requires many training samples, we collected mutation-drug-PMID triplets from PharmGKB [20]. PharmGKB provides manually curated mutation-drug relations with the ID of specific documents (PMID). Using this information, we collected mutation-drug relations from specific PubMed abstracts listed in PharmGKB, and found the sentences that mention both a mutation and a drug, as curated by PharmGKB. We used these sentences as the positive mutation-drug relation dataset. For the negative dataset, we found all the sentences that contain both mutation and drug names in PubMed abstracts. Among these sentences, we removed the sentences containing known mutation-drug relations that are contained in PharmGKB or BRONCO. Using this process, we collected 3133 sentences containing mutation-drug relations for the positive sentence-level dataset. We also sampled the same number of sentences from the pseudo-negative sentence set for the negative dataset.

#### Manually curated dataset for additional sentence-level evaluation

We also manually built and curated a dataset of sentences that contain mutation-gene and mutation-drug relations. After the list of PMIDs was filtered by PubTator, which is explained in the previous section, we found sentences containing at least one mutation and one drug for the mutation-drug sentence set, and sentences containing one mutation and one gene for mutation-gene sentence set. We automatically tagged mutations and gene names using BEST EE and randomly selected sentences from each sentence dataset. The sentence datasets were manually checked by two domain experts. Two curators classified relations as true or false in the sentence set. If the curators did not agree, the relations were discarded. The inter-annotator agreement score of the manual curation process is 68.1%. Finding mutation-gene relations is simple; however, classifying mutation-drug relations into binary classes is more complex. All the sentences in our manually curated evaluation set were annotated by at least two curators and we selected only the sentences on which both annotators agreed. The selected sentences were validated by a domain expert before they were included in the dataset. After this process, we collected 200 sentences for each positive and negative dataset. This dataset is used for the additional evaluation of the deep learning classification model which is trained on the PharmGKB mutation-drug sentence dataset.

#### Dataset from OncoKB actionable variant list for VarDrugPub evaluation

We collected mutation-drug data from OncoKB [40] which is a precision oncology knowledgebase that contains manually curated cancer-related mutation-drug relations. We collected only the single drugs with point mutation relations in the actionable variant list. From a total of 234 relations between point mutations and single drugs, we filtered the relations of drugs and mutations that were not mentioned together at the abstract level using PubTator. Finally, we collected 113 mutation-drug relations from OncoKB. We used this data for the qualitative analysis of our final results, which are combined in the VarDrugPub knowledgebase.

### Classification models using machine learning

For each evaluation, we trained machine learning classifiers such as decision trees, random decision forests and deep convolution neural networks (CNNs). We used Python version 2.7.10 with scikit-learn 0.17.0 as a decision tree and a random forest classifier machine learning tool. For the decision tree classifier, we followed all the hyper-parameter settings used in the method of Singhal et al., which is our baseline; otherwise, we used the default settings. We also used TensorFlow with Python for building deep learning classifiers.

#### Decision tree and random forest classifiers

A decision tree is also a well-known supervised-machine learning method used for classification and regression. It predicts the value of a target variable by decision rules using the data features of training data. Algorithms such as ID3 [41] or C4.5 [42] are widely used to build decision trees. Also, scikit-learn uses the optimized version of Classification and Regression Tree (CART) [43], which is based on C4.5, as its default algorithm to build decision trees for classification. Random forest is an ensemble learning method used for classification and constructing multiple decision trees in randomly selected subspaces of the feature space [43]. It can also be used to solve a decision tree classifier’s problem of overfitting the training data. In our evaluation, we mainly used a random forest classifier, which performed the best on our dataset. We used both the decision tree and random forest classifiers to evaluate the methods of Singhal et al. [26]; the authors claimed that the decision tree classifier worked the best in their evaluation.

#### Convolutional neural networks

We built a classification model using deep convolutional neural networks (CNNs). We modified the Tensorflow version of CNN sentence classification model of Kim [44, 45] to a CNN relation classification model. Most of the default settings and hyper parameters were remained as it was. We added position embedding, type embedding, BEST scores, and other features from the baseline methods.

**Fig. 3:**
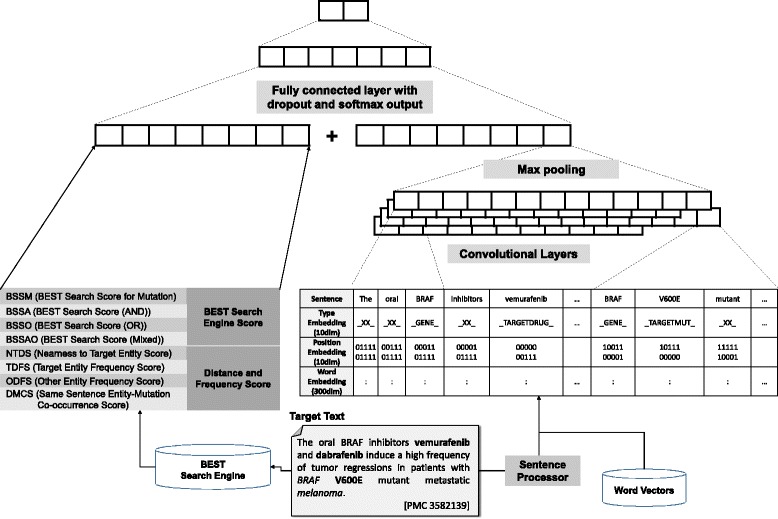
Relation classification model using deep convolutional neural networks

The process of sentence-level classification using CNNs and BEST scores is illustrated in Fig. 3. Each word in the sentences was embedded using pre-trained word2vec word vectors. Also, we added a 10-dimensional embedding vector of each word type (e.g., target mutation, target drug, target gene, genes, drugs and diseases that are not targets, etc.). We also added 10-dimensional embedding vectors that specify the relative position of words from each target entity [46]. We used TensorFlow version 0.8.0 for building our deep learning model [47].

**Fig. 4:**
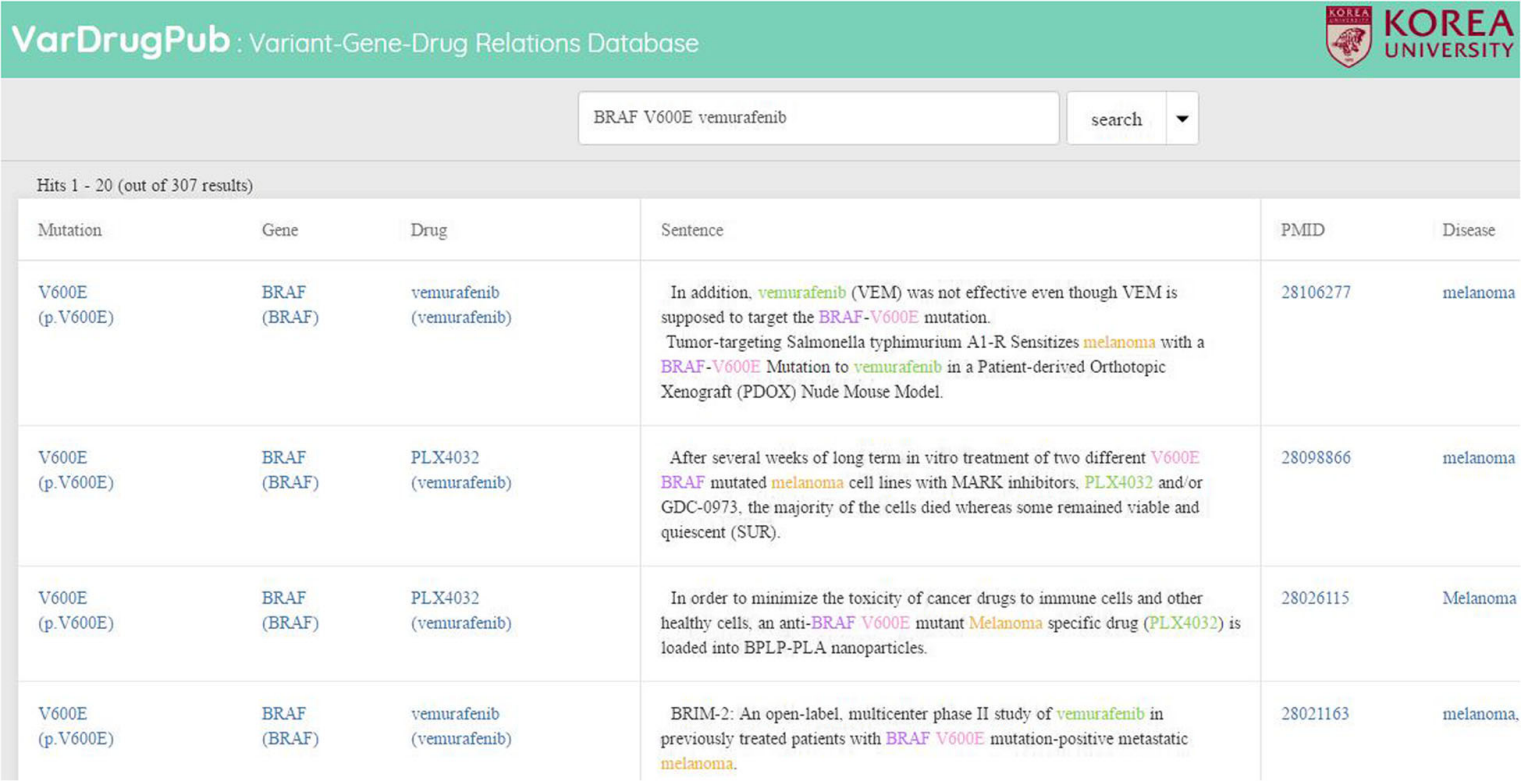
The result of search query “BRAF V600E vemurafenib” in our VarDrugPub database

## Results

### Evaluation methods

For the document-level model evaluation, we evaluated our models on the document-level dataset from BRONCO using 10-fold cross validation. In the set of document-level relation candidates, there is a substantial imbalance between the number of positive and negative data points. There were 277 positive and 9615 negative mutation-gene relations in the document-level dataset. There were 297 positive and 7658 negative mutation-drug relations. In each fold, we sampled the same number of positive and negative cases for training and testing. Since the number of positive relations is smaller, negative relations are randomly sampled to balance the ratio between positive and negative samples. To evaluate the performance of different methods, we used precision, recall and F1-score as the evaluation metrics.

In the sentence-level dataset evaluation of deep learning models, we obtained the average F1 scores after repeated five times of random sub-sampling validation. We did not use 10-fold cross validation because of the long training time of deep learning classifiers, and we wanted to use large amount of training data possible. For each repetition, we randomly selected 100 positive and 100 negative sentences as the test set and trained the model without the test set. Training sets are balanced so they have the same number of positive and negative cases, like the document-level dataset. In case there were more than two relations in the same sentence, we included a sentence only once to avoid overfitting.

### Baseline method

As it obtained the best result, we used the state-of-the-art method of Singhal et al. [26] as the baseline for document-level evaluation. Singhal et al. use not only the four frequency/distance scores introduced in Section 2.4, but also the two sentiment scores used in their methods. For the baseline results, we also included those two sentiment scores as features in the experiments. We used a C4.5 decision tree classifier with the same parameter settings that they used in their study. For both kinds of relations, random forest achieved better results than decision tree, as explained by the authors. It is important to note that their work is based on mutation-disease relations. Since we could not find any other mutation-drug relation classification model based on feature extraction and machine learning, we picked their method as our baseline. SNPshot [23] is designed to extract many relations between biomedical entities; however, it does not extract mutation-drug relations. The baseline method also worked greatly on our evaluation dataset and proved to be useful in finding mutation-gene and mutation-drug relations, as shown in Table 2.

**Table 2:** Results of relation mapping evaluation at the document level

Relation	Method*	Precision	Recall	F1-Score
Mutation-Gene	Decision Tree (Baseline features)	0.958	0.880	0.913
	Random Forest (Baseline features)	0.960	0.922	0.939
	Random Forest (Baseline features + search engine scores)	0.961	0.958	0.958
Mutation-Drug	Decision Tree (Baseline features)	0.796	0.787	0.788
	Random Forest (Baseline features)	0.798	0.820	0.806
	Random Forest (Baseline features + search engine scores)	0.830	0.819	0.821

*Baseline features: NTDS, TDFS, ODFS and DMCS from Singhal et al

*Search engine scores: BSSM, BSSA, BSSO and BSSAO

We did not compare the sentence-level result with the baseline models because the baseline models are designed for document-level extraction and require features that can only be extracted at document level. The baseline models’ performance at the sentence level will be lower than that at the document level, which makes the comparison unfair.

To evaluate the amount of “learning” achieved by our models, we evaluated an additional simple baseline representing “no learning” case. We performed co-occurrence-based predictions and report the results in Additional file 1: Table S2. In this analysis, we assume that when a mutation and an entity appear in the same text (i.e., sentence or document), they are classified as positive. The result of this no learning case is far inferior to our models, proving that our models “learn” complex non-linear relations among entities.

### Document-level classification

As shown in Table 2, our method for extracting mutation-gene relations achieved the best F1-score. One reason may be that the mutation-gene relations (e.g., BRAF V600E, V600E in BRAF) mentioned in text can be easily recognized by computational methods. Moreover, using the BEST search engine to find gene names associated with a mutation is very straightforward, as previously shown in the examples in Table 1. Mutation-gene relations are typically 1:1 relations, which means one mutation name is matched to a single gene name and they are usually mentioned together in an article.

Conversely, identifying mutation-drug relations is a different problem. One mutation can be associated with one or more drugs or none in an article. Mutation-drug relations do not have a clear pattern like mutation-gene relations; therefore, it is more difficult to find relations between them using traditional methods.

The mutation-gene results obtained by the baseline method of Singhal et al. were better than the mutation-drug results. In the BRONCO dataset, each mutation has only one associated gene; however, many mutation-drug relations are 1:n relations. In the baseline method, the three features NTDS, TDFS and ODFS are based on the closest distance (or the most frequently co-occurred) between target entities. If the relation is 1:1, we believe that the features used by the baseline method will work well as intended; however, if the relation is 1:n, the classifier might not train well, or only correctly identify the nearest relation. This may be the reason why the baseline method does not perform well in identifying mutation-drug relations.

### Sentence-level classification using word vectors, BEST scores and CNNs

As we have seen the importance of using BEST scores as classification features at the document-level, we also combined the scores in our deep learning model. We compared the classification results with the BEST scores and the results without the BEST scores for different word embedding sources. As shown in Table 3, the models using pre-trained word vectors achieved better results than models without pre-trained word vectors.

**Table 3:** Results of relation mapping evaluation at the sentence level with CNN. (The average F1-scores after five times of random sub-sampling validation)

Relation	Word2vec	Without BEST Scores	With BEST Scores
Mutation-Gene	None	0.943	0.947
	Google News	0.954	0.955
	PubMed (Token-based)	0.946	0.954
	PubMed (With BEST-EE)	0.941	0.951
Mutation-Drug	No word2vec	0.803	0.820
	Google News	0.845	0.864
	PubMed (Token-based)	0.829	0.841
	PubMed (With BEST-EE)	0.837	0.856

Interestingly, the model using the word vector trained on Google News achieved the best results. We believe Google News is a better source for training terms such as general verbs, general adjectives, and general nouns, while PubMed is a better source for training biomedical terms such as gene, disease and drug names. Even though we used word embeddings of biomedical entities in our deep learning model, the result of our deep learning models reflects that the general terms are more important than the embedding of biomedical entities in this relation classification task.

As also shown in Table 3, our deep learning model can use BEST scores as important features for classifying the relations. The results improved when the BEST scores were used as features, compared with when they were not used.

We added Additional file 1: Table S1 which provides details on the feature contribution analysis and Additional file 1: Figure S1 which illustrates the precision-recall curves in the Additonal file.

### Evaluation using manually curated sentences

We manually curated sentences containing mutation-drug relations for evaluation. We evaluated these sentences using the best-performing model which employs Google News word vectors and BEST scores. We obtained 0.871, 0.610 and 0.718 for precision, recall and F1-score, respectively. The difference in results of the two datasets is due to the difference in the guidelines. After error analysis, we found that in the manually curated dataset, the positive sentences contain many vague drug-mutation relations. Human curators classified them as positive; however, these unclear drug-mutation relations may not be very helpful for making a reliable dataset or knowledgebase for precision medicine. Our method is useful for collecting more definite relations as it obtains results with good precision.

### VarDrugPub: Mutation-gene-drug relation database

Finally, using the suggested deep learning method, we constructed VarDrugPub, a mutation-gene-drug relation database (Fig. 4). Utilizing PubTator, we collected all the PubMed abstracts that include at least one mutation and one drug name. In this filtered abstract set, we found all the sentences that contain both a mutation and drug name. Using our trained deep convolutional neural network model, we classified positive mutation-drug relations in the sentence set. We also found genes that are related to all the mutations that are found in this step using our classification model. Using results, we provide information about mutations, genes, drugs and the list of other biomedical entities that appear in the same document. It is possible to search the relations using single gene, drug or mutation names, and to use multiple terms as a query. All the identified mutation-gene-drug relations, the statistics of the data and further details are accessible on our website (http://VarDrugPub.korea.ac.kr).

### Evaluation of VarDrugPub using OncoKB dataset

VarDrugPub contains a total of 5712 unique mutation-drug relations. To qualitatively analyze our knowledgebase, we compared the mutation-drug relations in VarDrugPub with those in the OncoKB Actionable variant list. We considered only the single drugs with point mutation relations in the actionable variant list and those relations mentioned at the abstract level. Out of the 113 point mutation-single drug relations mentioned at the abstract level in OncoKB, 66 of them are also in our knowledgebase. We manually analyzed all the 47 relations that our method could not find but were included in OncoKB. 33 of the 113 relations did not co-occur at the sentence level, which cannot be detected by our sentence-level relation extraction model. 6 of the 113 relations were not clear. We could not find two relations due to the NER problem in the dataset generation process. Our model failed to detect the remaining 6 drug-mutation relations. We added these analysis results and details to the Supplementary file. These results demonstrate that our method can find many more mutation-drug relations than manual curation.

In this analysis, we realized the limitations of our method. We found that 121 of the OncoKB mutation-drug relations are not mentioned in abstracts. If we can utilize our method on full text, we can find many more new relations that are not mentioned at the abstract level. We also believe that if we extend our method from single-sentence level extraction to multi-sentence level extraction, we can find more missing relations in OncoKB.

## Discussion and conclusion

Here, we have proposed computational methods that automatically identify mutation-gene-drug relations in text using deep convolutional neural networks. Our deep learning model achieved better accuracy than the baseline methods. Our proposed methods also use the entire PubMed dataset to understand the existing relationships between entities. We used pre-trained word embeddings and entity search engine results to detect the relations between entities in PubMed abstracts. As demonstrated in the Results section, our methods use all the abstracts in PubMed database as background knowledge.

Our method that uses search engine scores is useful in finding the relations that are already mentioned together in existing publications. Even though there is no known relation between two entities, we use the other entities that are mentioned with the target entities in the same sentence to expand the query. These methods mimic how the manual curators use their background domain knowledge and the context of a text. We believe that the improvement in accuracy of our classification results prove that equipping machine learning tools with background domain knowledge is effective. However, if a mutation-gene-drug relation is novel and has not been previously reported in the literature, the search engine score will not be very helpful in finding the new relations between entities. In such cases, the extraction of the relations between the entities will depend more on the methods that focus on solely the text rather than outside knowledge. The suggested deep convolutional neural network models can detect these novel relations using pre-trained word embedding and numerous training examples.

Word vectors trained on other numerous texts such as Google News or PubMed abstracts represent another type of background information of entity-relations. Traditional biomedical text mining techniques used pre-defined keywords to explicitly describe the relations between entities [18, 23]. However, our approach does not require the manual selection of descriptive words. Instead, our approach learns relation words from other words using machine learning (CNN).

Most of the biomedical research studies on word vectors usually focus on biomedical entity terms [35, 48, 49]. However, we used word vectors with CNNs to find relations between entities. Our novel deep CNN models with word embedding and entity search scores can be readily used in other applications.

We also observed that in our task, the Google News word vector obtained better results than the PubMed word vector. We expected that PubMed would be better for biomedical entity relation classification; however, we believe that general terms such as verbs and adjectives are more important than biomedical entities in this task of describing the relations between the entities. Additional experiments are required to explain which word vectors are better for biomedical text mining tasks. We still believe, since we build word vectors for all the words that appear in PubMed, the words are optimized for biomedical text mining in PubMed. We leave this problem for future work.

In this research, we used only CNN as our deep learning classifier; however, we believe we can extend this study using other deep learning models such as recurrent neural network or recursive neural network. We will leave this problem for future work as well.

We observed that not all the mutation-drug relations can be explained in single drug – single mutation relation. Drug combination research studies or other studies on the effect of multiple mutations require multiple drugs or multiple mutation relation extraction methods. Our method needs to be expanded to combination of multiple entity relation extraction. We also believe that using better NER tools will improve knowledge extraction.

In conclusion, we have developed a set of novel computational deep learning methods that integrate search engine scores and word embedding for identifying mutation-gene and mutation-drug relations in text. The methods utilize background knowledge in PubMed abstracts as features for machine learning classifiers. We demonstrated that using the PubMed database as background knowledge improves the classification results. To the best of our knowledge, our approach is the first that combines biomedical entity search and word embedding using deep learning to utilize background knowledge for mutation-entity relation extraction from the literature.

## Additional file

Additional file 1: Table S1. Feature contribution analysis in our CNN model. **Table S2**. Results of simple co-occurrence-based method. **Table S3**. VarDrugPub and OncoKB comparison examples. **Figure S1**. Precision-Recall curves of our CNN classifier (Blue: Mutation-Gene, Red: Mutation-Drug). (DOCX 50 kb)

## Abbreviations

BEST EE: BEST (Biomedical Entity Search Tool) Entity Extractor
CNN: Convolutional Neural Networks
DMCS: Disease (or Drug)-mutation Co-occurrence Score
NER: Named-Entity Recognition
NTDS: Nearness to Target Disease Score
ODFS: Other Disease Frequency Score
TDFS: Target Disease Frequency Score
