# Supplementary material for: Deep learning of mutation-gene-drug relations from the literature
Source: BMC Bioinformatics. 2018 Jan 25;19:21. doi: 10.1186/s12859-018-2029-1 (PMC5784504; doi:10.1186/s12859-018-2029-1)
Supplement: Additional file 1: Table S1. — Feature contribution analysis in our CNN model. Table S2. Results of simple co-occurrence-based method. Table S3. VarDrugPub and OncoKB comparison examples. Figure S1. Precision-Recall curves of our CNN classifier (Blue: Mutation-Gene, Red: Mutation-Drug). (DOCX 50 kb) [file 12859_2018_2029_MOESM1_ESM.docx]

Supplementary File

Deep Learning of Mutation-Gene-Drug Relations from the Literature

Kyubum Lee^1,†^ ,Byounggun Kim^2,†^ ,Yonghwa Choi^1^, Sunkyu Kim^1^, Wonho Shin^1^, Sunwon Lee^1^, Sungjoon Park^1^, Seongsoon Kim^1^, Aik Choon Tan^3,*^ and Jaewoo Kang^1,2,*^

*1. Department of Computer Science and Engineering, Korea University, Seoul, Korea.*

*2. Interdisciplinary Graduate Program in Bioinformatics, Korea University, Seoul, Korea.*

*3. Translational Bioinformatics and Cancer Systems Biology Laboratory, Division of Medical Oncology, Department of Medicine, University of Colorado Anschutz Medical Campus, Aurora 80045, CO, USA.* [aikchoon.tan@ucdenver.edu](mailto:aikchoon.tan@ucdenver.edu) and [kangj@korea.ac.kr](mailto:kangj@korea.ac.kr)

*To whom correspondence should be addressed.

^†^ These authors contributed equally to the work

1. Feature Contribution Analysis

In this research, we used four different features as explained in Section 2.3.2. To measure the contribution of each score on our deep learning classifier, we performed additional experiments.

We used the same method that was explained in Section 2.7 to train our CNN classifiers, but we shuffled the values of features in the test set. For example, to test the effect of BSSM feature, we shuffled all the BSSM scores in both positive and negative data in the test set, and re-classified the test set. The results are shown in Table S1. We trained 5 classifiers using the same setting and tested them on a holdout set. Google News word vectors were used for word embedding.

From this result, we can observe that BEST scores play a more significant role in mutation-gene classification than mutation-drug classification. Frequency scores are comparably more important in mutation-drug classification.

Table S1. Feature contribution analysis in our CNN model

| **Relation** | **Shuffled Feature** | **Average F1 Score*** |
| --- | --- | --- |
| Mutation-Gene | No features are shuffled | 0.961 |
|  | Frequency scores | 0.957 |
|  | All the BEST scores | 0.917 |
|  | BSSM | 0.939 |
|  | BSSA | 0.947 |
|  | BSSO | 0.960 |
|  | BSSAO | 0.941 |
| Mutation-Drug | No features are shuffled | 0.857 |
|  | Frequency Scores | 0.835 |
|  | All the BEST scores | 0.833 |
|  | BSSM | 0.840 |
|  | BSSA | 0.854 |
|  | BSSO | 0.851 |
|  | BSSAO | 0.842 |

* The average of F1 scores obtained from five models

**2. Precision-Recall curve of the classification results.**

Figure S1 shows the Precision-Recall curves of our best performing model (Blue: Mutation-Gene, Red: Mutation-Drug).


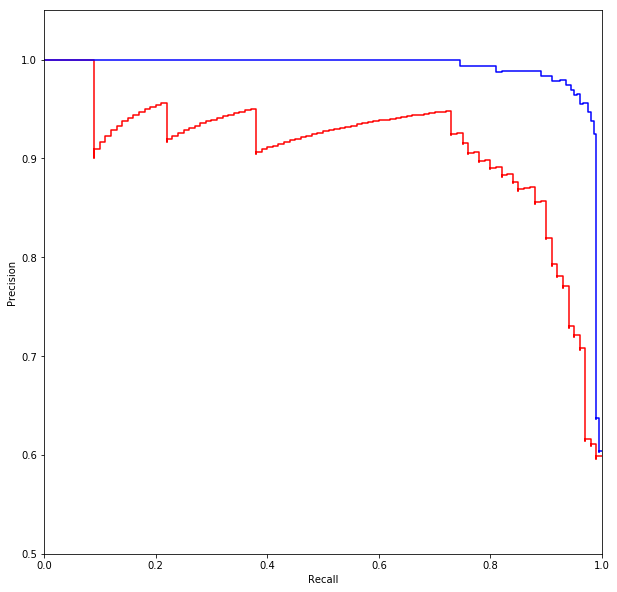


Figure S1. Precision-Recall curves of our CNN classifier (Blue: Mutation-Gene, Red: Mutation-Drug)

**3. Co-occurrence analysis results**

As a simple baseline representing “no learning” case, we report the results of a co-occurrence-based method. Table S2 shows the co-occurrence analysis results at the sentence and document level. In this analysis, we assume that when a mutation and an entity are at the same sentence/document level, they are classified as positive and compared with the gold standard results.

For sentence level precision, we used the statistics obtained from the manual curation process.

For document level precision, we used the dataset BRONCO (Lee et al., *Database* 2016). We obtained Mutation-Gene-Drug triplets from BRONCO. We generated all the possible mutation-gene and mutation-drug pairs in each document, and compared them with the triplets from BRONCO.

Note that recall is not shown in Table S2 because the recall of this method is always 100% as the method returns all the possible candidate answers as the prediction results.

As shown in the results, our methods perform far better than simple co-occurrence-based methods, proving that our models “learn” complex non-linear relations among entities.

Table S2. Results of simple co-occurrence-based method

| **Relation** | **Shuffled Feature** | **Precision** | **F1** |
| --- | --- | --- | --- |
| Mutation-Gene | Sentence level  (Manual curation) | 0.609 | 0.757 |
|  | Full text level  (BRONCO) | 0.499 | 0.666 |
| Mutation-Drug | Sentence level  (Manual curation) | 0.332 | 0.498 |
|  | Full text level  (BRONCO) | 0.446 | 0.617 |

**4. VarDrugPub and OncoKB Comparison analysis**

VarDrugPub version: May 23, 2017

OncoKB Actionable Variants version: August 20, 2017 - (Single drugs, point mutations only)

Total number of unique Mutation-Drug relations in VarDrugPub: **5,712**

Total number of unique single point mutation- single drug relations in OncoKB: 234

Total number of unique single point mutation- single drug relations **co-occurred at the abstract level** in OncoKB: **113**

Overlapped Mutation-Drug relations: **66**

- VarDrugPub only relations: **5,646**
- OncoKB only relations: **47**
  - Do not co-occur in sentence-level: **33**
  - Not very clear, or not strong relations: 6
  - Named-entity recognition error: 2
  - Others: 6

Table S3. VarDrugPub and OncoKB comparison examples

| **Type of  error** | **Target Mutation** | **Target Drug** | **PMID** | **Sentence** |  |
| --- | --- | --- | --- | --- | --- |
| NER Error | D816F | dasatinib | 16397263 | Furthermore, dasatinib is a potent inhibitor of imatinib-resistant KIT activation loop mutants and induces apoptosis in mast cell and leukemic cell lines expressing these mutations (potency against KIT D816Y>> D816F > D816V). |  |
| NER Error | E17K | azd5363 | 28489509 | The genomic context of the AKT1 E17K mutation further conditioned response to AZD5363. |  |
|  |  |  | 28472036 | Both assays are employed at centralized testing laboratories operating according to quality standards for prospective identification of the AKT1 E17K mutation in ER+ breast cancer patients in the context of a clinical trial evaluating the AKT inhibitor AZD5363 in combination with endocrine (fulvestrant) therapy. |  |
|  |  |  | 26931343 | Akt1 (E17K) is a potent driver mutation that may predict clinical response to AZD5363. |  |
| Not clear / not strong relations | W557G | imatinib | 23567324 | Both the in-silico/in-vitro investigations showed constitutive activation and sensitivity to Imatinib of the yet mentioned Y578C mutation as well as of the double mutant, providing evidence that the concomitant presence of the W557G and Y578C mutations does not affect Imatinib response compare to the single mutations, in line with what observed in Imatinib treated patient. |  |
|  | D2033N | cabozantinib | 26673800 | In contrast, cabozantinib binding was unaffected by the D2033N substitution, and inhibitory potency against the mutant was retained. |  |
|  | V560D | imatinib | 20633291 | Motesanib also demonstrated activity against kinase domain mutations conferring imatinib resistance (V560D/V654A, IC50 = 77 nM; V560D/T670I, IC50 = 277 nM; Y823 D, IC50 = 64 nM) but failed to inhibit the imatinib-resistant D816V mutant (IC50 > 3000 nM). |  |
|  | E709K | Gefitinib, erlotinib | 27323238 | For example, G719X (X denotes A, S, C and so on), Del18, E709K, insertions in exon 19 (Ins19), S768I or L861Q showed moderate sensitivities to gefitinib or erlotinb with ORR of 30%-50%. |  |
|  | L576P | nilotinib | 28327988 | Similar to previously reported results with imatinib, nilotinib showed greater activity among patients with an exon 11 mutation, including L576P, suggesting that nilotinib may be an effective treatment option for patients with specific KIT mutations. |  |
|  |  |  | 19671763 | In vitro testing showed that the cell viability of the L576P mutant cell line was not reduced by imatinib, nilotinib, or sorafenib small molecule KIT inhibitors effective in nonmelanoma cells with other KIT mutations. |  |
| ETC | D846Y, N848K | imatinib | 15928335 | Interestingly, other mutations in exon 18 (D846Y, N848K, Y849K and HDSN845-848P) were all imatinib sensitive. |  |
|  | N822I | dasatinib | 21689725 | In addition, we demonstrated that KIT-N822I is resistant to imatinib and sensitive to dasatinib. |  |
|  | V559D | sorafenib | 17699867 | The Ba/F3KIT(WK557-8del/T670I) cells were sensitive only to sorafenib inhibition, whereas nilotinib was more potent on imatinib-resistant KIT(V560del/V654A) and KIT(V559D/D820Y) mutant cells than dasatinib and sorafenib. |  |
|  | H697Y | sunitinib | 19861435 | In cell viability assays, the V560del mutant was associated with similar sensitivities to imatinib and sunitinib, whereas the H697Ymutant displayed greater sensitivity to sunitinib. |  |
|  | V560D | sunitinib | 20095048 | This result correlates with the V560D mutant exhibiting a sensitivity to sunitinib that is less than for WT KIT but greater than for KIT D816H |  |
